# Evaluating Usability and Feasibility of Implementing a Novel Cancer Mapping Tool

**DOI:** 10.21203/rs.3.rs-5321299/v1

**Published:** 2024-11-15

**Authors:** Erin Wissler Gerdes, Jinyi Cai, Carly Mahoney, Grant Brown, Jacob Clark, Mary Charlton, Caglar Koylu, Emily Roberts, Brittany McKelvey, Charles Wiggins, Angela Meisner, W. Jay Christian, Bin Huang, Jacob Oleson, Sarah Nash

**Affiliations:** University of Iowa; University of Iowa; University of Iowa; University of Iowa; University of Iowa; University of Iowa; University of Iowa; University of Iowa; University of New Mexico; University of New Mexico; University of New Mexico; University of Kentucky; Markey Cancer Center; University of Iowa; University of Iowa

**Keywords:** cancer registry, small area cancer incidence, Bayesian, cancer prevention and control

## Abstract

**Purpose:**

Cancer registries are often asked to present cancer data for small geographic areas to inform and facilitate targeted interventions and prevention programs. However, it is challenging to compute and visualize reliable cancer estimates for areas with small case counts and populations to support cancer control planning.

**Methods:**

We used a Bayesian hierarchical model that borrows strength from neighboring areas and over time to produce cancer estimates for small areas. We developed a visual analytics platform to present these estimates in interactive graphics that demonstrate risk in small areas. In a user-centered design process, development of the tool was informed by cancer registry and public health professionals through focus groups and surveys.

**Results:**

The Cancer Analytics and Maps for Small Areas tool (CAMSA) provides age-adjusted cancer incidence and mortality rates and risk probabilities for eight cancers at the county and ZIP-code tabulation area (ZCTA) levels. It allows the user to identify cancer hotpots, including among sub-groups defined by sex and race/ethnicity. Potential end users were enthusiastic about the opportunity to implement CAMSA within their practice, emphasizing the tool’s potential for increasing collaborative opportunities at local and state levels. Suggestions for improvement included adding map overlays such as additional cancer risk variables and incorporating functionalities like exportable data tables.

**Conclusions:**

CAMSA presents cancer rate and risk estimates for small geographic areas where they may have previously been suppressed. Through our user-informed design process, we developed statistical models and data visualizations to support the needs of an array of potential end users.

## INTRODUCTION

Many cancers with modifiable risk factors have higher incidence in rural populations compared to urban populations [1]. Furthermore, cancer-specific mortality rates are decreasing at faster rates among urban versus rural residents [2]. When considering the connectedness of characteristics such as rurality and race/ethnicity, additional patterns in both incidence and mortality emerge, and vary by cancer site [3, 4]. In order to better support local-level interventions that will reduce cancer risk among specific populations in small geographic areas, incidence and mortality rates need to be reported at the most granular level possible. However, it is challenging to accurately assess cancer risk in small geographic areas, especially in the case of low-incidence cancers [5], or among sub-populations (e.g., strata of age, race/ethnicity, sex). Standard epidemiological methods using small case counts can produce unreliable estimates for disease risk [6], and small case counts may result in data suppression.

Previous work modeling Iowa Cancer Registry data used a Bayesian modeling approach including spatial correlation, which helps smooth unreliable and unstable estimates by borrowing strength from neighboring geographic areas while avoiding the potential identification of individual cases [7]. Yet, several gaps remained, including the inability to stratify by factors of potential interest, a lack of process to develop these models for use beyond Iowa registry data, and the lack of a data visualization platform to disseminate results. Thus, this project aimed to create a tool to generate and visualize estimates for cancer incidence and mortality rates for small areas, currently county and ZIP code tabulation areas (ZCTA), across strata of sex, race/ethnicity, and diagnosis year [7, 8]. Through the use of a user-centered design process, we developed the Cancer Analytics and Maps for Small Areas (CAMSA) tool to be applicable across multiple audiences, and to have the requisite infrastructure to broaden its reach beyond the initial test states of Iowa (IA), Kentucky (KY), and New Mexico (NM). Here, we present the process of developing CAMSA, and describe potential uses across cancer surveillance, prevention, and control.

## METHODS

### Study Design and Setting

This project was led by investigators at the University of Iowa, in collaboration with colleagues at the University of Kentucky and University of New Mexico. All data were from the National Cancer Institute’s Surveillance, Epidemiology, and End Results (SEER) Cancer Registries, including the Iowa Cancer Registry, the Kentucky Cancer Registry, and the New Mexico Tumor Registry.

CAMSA development was conducted using a user-centered design process, which involves collecting feedback from target users throughout the design and development of the interface [9]. Development was conducted within the user-centered design *user→ utility→ usability* feedback loop structure, where target users first detail needs from the tool (user), then revisions are made to the conceptualization and functional requirements of the tool (utility), and then new tool iterations are created (usability) [9]. Within every stage of this development mechanism, end users provided feedback through focus groups and/or surveys; future activities will include usability testing. Herein, we describe our process through the development of CAMSA versions 1.0 through 2.0.

#### Small Area Statistical Analysis

Using Bayesian hierarchical modeling described previously [7], small area estimates were computed at both the ZIP code tabulation area (ZCTA) and county level for eight cancer categories (colorectal [ICD-O codes: C180–189, C260], female breast [C500–509], cervical [C530–539], liver [C220], lung and bronchus [C340–349], melanoma [C440–449], prostate [C619], and non-Hodgkin’s lymphoma [C024, C098, C099, C111, C142, C379, C422, C770–779], based on ICD-0–3/WHO 2008 site recode) [10] with modifiable risk factors and screening modalities. ZCTA was chosen for the initial development of the tool as it was considered easily understandable by the general public, one of our pre-defined end user populations. Additional parameters for modeling were outcome (incidence, late-stage incidence, or mortality) and year or demographic stratification (sex, year range, race, or sex and year range).

We estimated multiple measures of cancer risk, including age-adjusted rates per 100,00 population and risk probabilities. Risk probabilities indicate the relative degree of risk for each ZCTA/county compared to the state average, so a value of 95% represents a high probability of cancer burden that is greater than the overall state. All modeling was completed in R using the NIMBLE package [11, 12].

We utilized different processes for conducting modeling and creating maps for partner registries, in order to develop a choice of processes that might be scalable to other central cancer registries. The New Mexico Tumor Registry sent registry data to the University of Iowa to create cancer estimates and maps for New Mexico under a data use agreement. The University of Kentucky utilized a University of Iowa-developed R package to run their own estimates from registry data. The CAMSA interactive web tool was developed using JavaScript libraries including Bootstrap for responsive interfaces, Leaflet for map visualization and Chart.js for displaying charts.

#### User-centered Design

**Figure 1:**
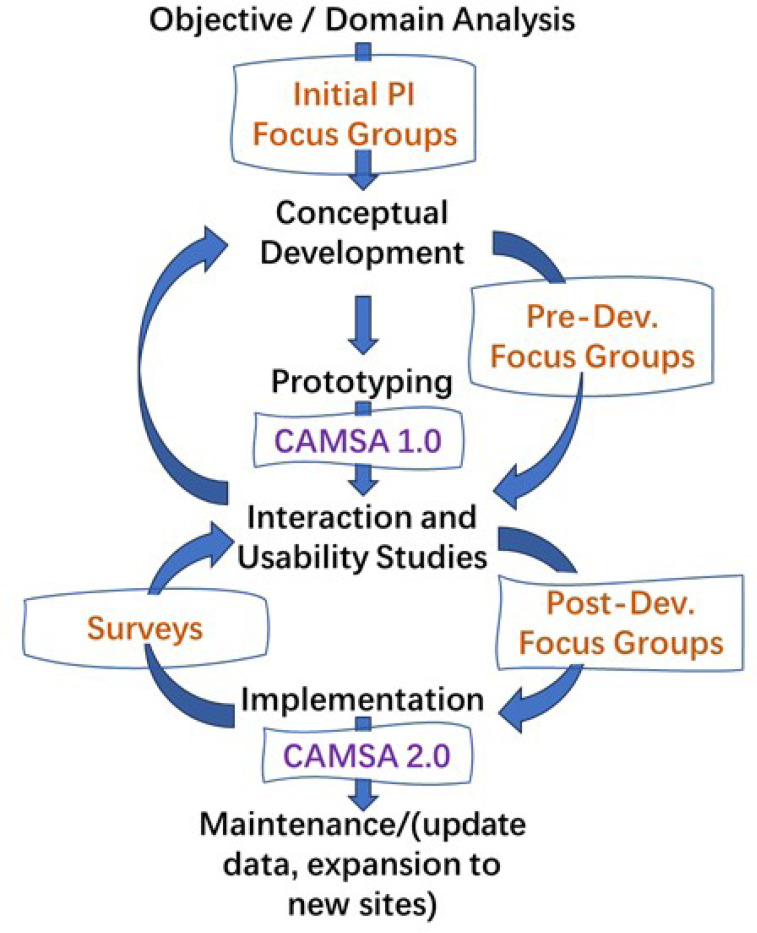
User-centered design structure showing how user feedback was incorporated throughout each step of the process.

**Figure 2:**
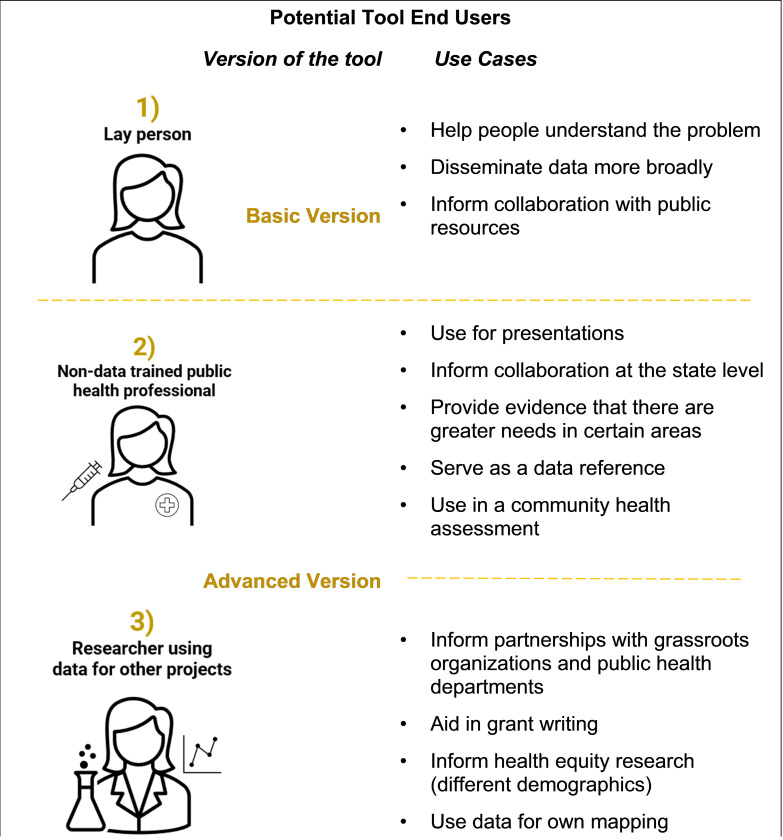
Three end users with specific use cases were identified in focus groups.

We constructed an iterative user-centered design framework (see Fig. 1) based on works from Roth et al. [9] and Robinson et al. [13] that informed the development of CAMSA. The objective of following the iterative user-centered design process was to ensure CAMSA would provide relevant data in an approachable, interactive visualization format to a variety of potential end users. Figure 2 also shows how user feedback was incorporated at every step of the conception and design of CAMSA:

##### (1) Conceptual Development

Two members of our team (EWG, SHN) met with registry-based investigators from IA, NM and KY to discuss potential end-users of CAMSA and identify individuals within their states that held relevant roles in cancer prevention and control. Potential end users identified through this process were invited to participate in focus groups, as described below.

##### (2) Prototyping

We conducted two pre-development focus groups with four participants each, and seven key informant interviews across the three states via video calls from October 2022 through January 2023. Questions (Supplementary Table 1) focused on whether individuals (or their workplaces) would use this tool; how it might be used; features that they’d like to see included, etc. This study led to the formalization of core functional requirements: 1) data filtering, 2) map design and interaction, 3) data table and charts, and 4) data and map exporting. The initial version of CAMSA (CAMSA 1.0) was developed based on these requirements.

##### (3) Interaction and Usability Studies

After the development of CAMSA 1.0, we invited the same end users from IA, NM, and KY to participate in additional focus groups to elicit preliminary feedback on the tool (Supplementary Table 1). After a brief demonstration of the tool, questions focused on users’ initial responses, likes, dislikes, and identified gaps. In total, we conducted seven focus groups with 23 participants across the three states. Focus groups were conducted via video call from November 2023 through February 2024. For each set of focus groups, we conducted a rapid thematic analysis,[14, 15] and findings were discussed in internal meetings and shared via reports to the design team to inform tool development. Findings of these focus groups were incorporated into the second iteration of the CAMSA tool (CAMSA 2.0).

##### (4) Implementation

In order to assess central cancer registry capacity, interest, and potential use cases for this work more broadly outside of Iowa, New Mexico and Kentucky, we conducted a ten-item survey (Supplementary Table 2) at two separate national meetings (North American Association for Central Cancer Registries Annual Meeting, June 2024; and the SEER Research Meeting, July 2024). The tool was presented in brief, and audience members were invited to complete the survey to quantitatively assess interest in using the tool; ways in which the tool would be used; registry capacity to administer the tool (i.e., capacity to run the R program in-house versus share data with the Iowa team); and solicit additional feedback. We conducted descriptive analyses of the 17 survey responses using Qualtrics software (Qualtrics, Provo, UT).

## RESULTS

### Focus Group Feedback on Conceptual Development Potential End Users and Use Cases

Registry-based investigators identified end-users to consider during tool development including researchers in academia, public health practitioners, and individuals working with and for cancer-related nonprofit organizations. Representatives from these domains from across IA, KY, and NM were invited to participate in focus groups.

Focus group participants identified three potential end users for the tool, including 1) a lay person with personal interest in cancer and/or patient advocate, 2) a non-data trained public health professional, and a researcher using data estimates to support grant applications or research projects. Figure 2 shows these end-users and their potential uses for the tool, as described by our focus group participants. Specific use cases identified for CAMSA included highlighting cancer clusters and hotspots for public health initiatives; supporting community health needs assessments; as a public-facing public health data source; and, as a basis for partnering with other organizations directly or indirectly involved in cancer prevention and control.

### CAMSA Version 1.0

**Figure 3:**
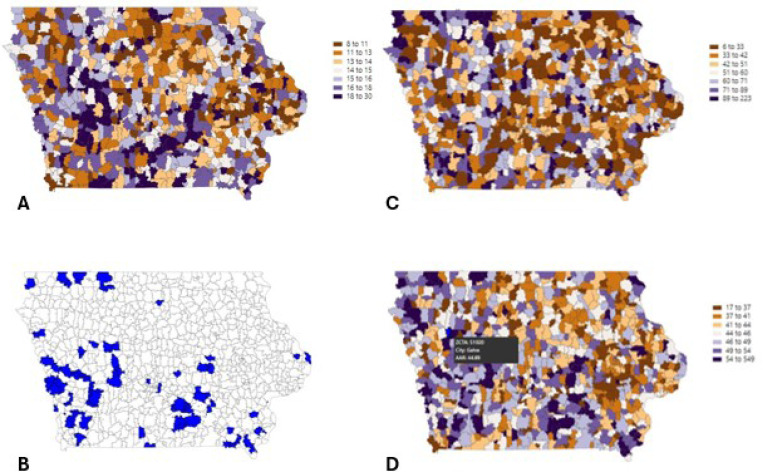
**A:** Age-adjusted mortality rates of colorectal cancer at the ZCTA level in the state of Iowa as displayed in CAMSA v1.0. The color scale highlights higher rates (orange) and lower rates (purple) across the state. **3B:** top 90^th^ percentile of Iowa ZCTAs of age-adjusted colorectal cancer rates. **3C/3D:** Age-adjusted colorectal cancer rates at the ZCTA level in Iowa stratified by race. **3C** shows Black colorectal cancer incidence rates and **3D** shows White colorectal cancer age-adjusted incidence rates.

CAMSA 1.0 presented age-adjusted rate and risk probability estimates for eight cancer types (colorectal, breast, cervical, liver, lung, melanoma, non-Hodgkins’s lymphoma, and prostate). Estimates were viewable at either county or ZCTA level in 23 different color schemes. For example, Fig. 3A shows age-adjusted cause-specific mortality rates of colorectal cancer at the ZCTA level in Iowa.

Another key feature of the tool was the ability to highlight hotspots or clusters of cancer incidence. Figure 3B shows a map indicating the ZCTAs having the top 10% rates of colorectal cancer incidence in Iowa. Additionally, the user could stratify by different variables, including race and ethnicity. As shown in Figs. 3C and D, ZCTAs with higher colorectal cancer incidence rates are different among Iowa’s Black and White populations, respectively.

### Focus Group Feedback on CAMSA Version 1.0

Focus group feedback was focused on the following themes: useful features and suggestions for improvement, use cases for CAMSA, implementation and training needs, and concerns about implementing CAMSA.

### Useful Features and Suggestions for Improvement

Responding to CAMSA 1.0, focus group participants appreciated language on how to interpret results; however, they identified a need to tailor explanatory language to each end user group (i.e., the lay public versus public health professionals). Most participants stressed the importance of using clear and concise language, including developing a tool glossary, hover box descriptions for each tool button, and generated map titles.

Participants found it useful to search by county or ZCTA and filter by different demographics; although, some individuals expressed a desire to model census tracts, which are more commonly used by researchers. Participants wanted both the created map and accompanying data table available as exportable files to incorporate into grants, presentations, and other data visualization programs. The option to have accessible color scale options on the map was appreciated, but participants found the 23 options overwhelming.

Finally, participants suggested creating a basic and advanced version of the tool tailored to the needs of the different end user populations. They suggested that the basic version might include simple estimate calculations with fewer filter options, whereas the advanced option might include overlays with public health regions and significant landmarks, as well as more advanced variable stratification and options to download estimates to export into alternate visualization tools.

### Uses for CAMSA

Feedback from both the focus groups and surveys indicated that CAMSA would foster collaboration. Focus group participants discussed potential collaboration at the state level due to the tool showcasing evidence of cancer needs in specific geographical areas. Additionally, participants noted that having a public-facing data platform would allow for increased communication with the public around cancer concerns and hot spots.

### Implementation

Focus group participants and survey responders identified several types of training and support that would be beneficial in implementing this tool. Video tutorials were identified as the most helpful training tool, in addition to other visual-based training, including infographics and screenshots of the tool with step-by-step instructions.

### Concerns about CAMSA

There were several potential challenges identified by focus group participants. Some potential areas for concern included people not understanding the estimates or misinterpreting the data; for example, there was concern that the data points calculated by the tool would be interpreted as exact values rather than statistical estimates. Additionally, there was concern that the private sector would not want the public to associate cancer clusters with private organizations if overlays of significant landmarks were to be incorporated with the tool.

### CAMSA Version 2.0

**Figure 4:**
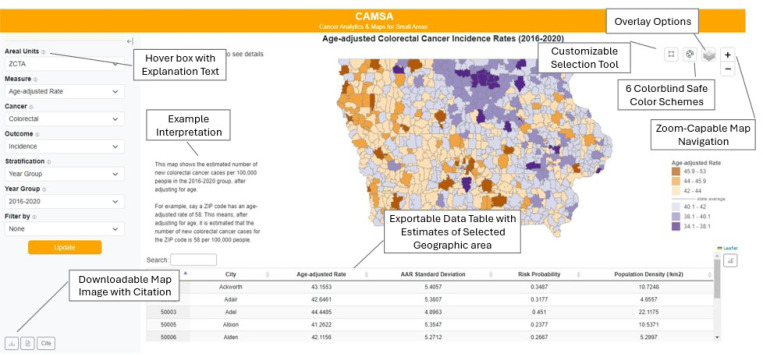
CAMSA 2.0 with features incorporated from user feedback. Tool additions are annotated.

CAMSA version 1.0 was substantially revised in response to information obtained from the focus groups. Priorities were categorized into functional requirements, or the necessary tasks, actions or activities that must be accomplished. The functional requirements encompassed four items: 1) data filtering, 2) map design and interaction, 3) data table and charts, and 4) data and map exporting. Figure 4 shows an annotated version of CAMSA 2.0 highlighting where these elements were incorporated.

The filtering options from CAMSA 1.0 were retained with conditional display features; for example, selecting prostate for the cancer type option automatically hides the female sex group option to prevent displaying null data. Additionally, hover boxes with explanatory text were added to help users quickly understand unfamiliar terminologies. When users update data filter options, data on map and table change dynamically.

Essential map elements including legends, title, hover box and explanatory text were re-designed to improve clarity. Color schemes were reduced to six color-blind-friendly color schemes for age-adjusted rate and risk probability measures. Additionally, users can select areas of interest using the new customizable selection tool to compare the statistical measures. Additional layers including health planning areas, Superfund sites [16], and major cities can be overlaid on the map to provide further context. To indicate the uncertainty of the statistical results, standard deviations for age-adjusted rates were classified into low, medium and high levels of uncertainty based on the quantile values, and presented in an overlay option.

Data tables and maps were dynamically linked so users could click a table row to highlight corresponding map areas and vice versa. A new search box was integrated into the table, allowing users to specify areas of interest using ZCTA codes, county names, city names, or values such as age-adjusted rates, risk probabilities, and population density.

The data and map exporting function was implemented to allow users to export the current map view as a .PNG file and the table of data estimates as a .CSV file. The exported map includes the map source, enabling audiences to easily trace back to the CAMSA platform.

To increase usability of CAMSA, we developed a flexible interface allowing users to explore data filtering, map, and chart modules. Users can collapse the sidebar after filtering or click a button to display charts in a dialog box. We also added a map explanation section to clarify the measures with selected area unit, cancer type, outcome type, stratification group, and filter status. This section also includes an example interpretation to assist users unfamiliar with summarizing and explaining cancer data.

### Survey Results

We received 17 total survey responses in response to our demonstration of CAMSA 1.0 at the North American Association of Central Cancer Registries (NAACCR) Annual Meeting (June 2024), and SEER Research Meeting (July 2024). In response to questions on potential uses of CAMSA, respondents reported that they would use the tool to highlight hot spots or clusters (71%), support community health or public health assessments (71%) and to support presentations or grant writing (53%). Many of the respondents were receptive to the idea of CAMSA being integrated into their practice, though there was a difference in capacity for each registry and how the tool would be implemented. While 65% reported that they had the capacity to implement the tool into their practice, 35% were unsure if they had the capacity. Respondents had a range of registry capacity to implement this tool, with most reporting capacity in biostatistics (67%), testing and usability (58%), and administrative capability (50%). Overall, most respondents were interested in implementing this tool in their registry (65%) and collaborating in the future (69%).

## DISCUSSION

In this report, we present a novel, web-based tool that can be used by cancer registries to generate small area statistics for the areas/populations they serve, and describe the user-centered design process used to develop the tool. The tool uses Bayesian hierarchical modeling to produce estimates for incidence, late-stage incidence, and mortality for eight common cancers in small geographic areas, including ZCTA and county level; future iterations will also include census tract modelling. Participants identified several use cases for this tool based on user experience. Lay users might use the tool to better understand the existing state of cancer impacts and issues in their community. Advanced users could use the tool to support public health initiatives targeting clusters, as well as for fostering collaboration with grassroots organizations, local healthcare providers, and public resources. In summary, user feedback suggests that this novel tool will provide a useful resource for central cancer registries to compute cancer risk data at small geographic area levels that are often suppressed due to small case counts and/or population sizes. This will allow collaboration with cancer prevention and control professionals to design and implement targeted initiatives to lower cancer risk in small (rural) communities.

A user-centered design process was central to the successful development of CAMSA 2.0. User-centered design approaches have been widely used as a process for developing interactive tools.[9, 17, 18] In the field of cancer epidemiology, user-centered design has effectively been utilized to evaluate the usability of several data visualization tools, including the Pennsylvania Cancer Atlas and the Exploratory Spatio-Temporal Analysis Toolkit, both of which incorporated user feedback in the design and implementation of data visualization [13, 19]. In both cases, the feedback from visualization and usability experts helped improve cartographic and interface design, and feedback from cancer control experts helped assess and promote the utility of the tool for the target end user [13, 19].

Through this user-centered design process, focus group participants and survey respondents helped highlight the useful features of the tool and suggested improvements for future iterations. The current stratification options, including race and ethnicity, sex, and year, enable users to fine tune data estimates for specific population groups. Furthermore, the estimates are shown in both an interactive map and a table to facilitate clear and intuitive interpretation. Subsequent iterations include the ability to overlay landmarks on the map for the user to orient the county and ZCTA level data, adjusted color scale options; interpretation and user guides for different end users; and exportability of data estimates. One of the most requested items for future development was the addition of other kinds of data, including area-based social measures, environmental data, and cancer screening locations. These additional data components will be considered for future iterations of the tool.

Existing tools, like Cancer InFocus [20], NCI Cancer Zones [21], and CDC Places [22], have similar use cases, but certain features make CAMSA uniquely useful for surveying small area cancer data. Cancer InFocus and NCI Cancer Zones provide data at the county level or larger, and while CDC Places provides data at the ZCTA and census tract levels, it does not have cancer-site data available. Our project combines the best features from each of these existing tools, while employing novel modeling methods to produce estimates for eight cancers. Furthermore, the tool meets the needs of end users, and our surveys indicated that central cancer registries have at least some capacity to implement this new tool through the mechanisms already developed (i.e., running R code for their own registries, or sharing data with the University of Iowa for our team to run models and develop the interface). While CAMSA has many strengths, continued development is needed to ensure that this tool is maximally responsive to end-user needs. Additionally, many of the suggested improvements (e.g., overlaying environmental data and other cancer risk factor variables) will be incorporated in the next iteration of this tool.

## Conclusion

The goal of this project was to create a tool that can be used by central cancer registries to visualize their data and support public health practitioners in their state to identify cancer hotspots and inform cancer prevention and control planning. This user-centered design process helped our team understand the full spectrum of use cases for this tool and identify three types of end users (general public, public health professionals, data users). Further, we were able to identify favorable features of the tool and prioritized items for future iterations. Overall, our focus group and survey feedback indicate that CAMSA will meet the needs of cancer registry and public health users, and suggested adjustments could help maximize its utility in facilitating cancer prevention and control initiatives for small geographic areas.

## Supplementary Material

Supplement 1

## Data Availability

Cancer registry data utilized in this project are available by application to each SEER cancer registry. Survey and focus group data reported herein are available by reasonable request to the corresponding author. R programs are available through GitHub through request to the corresponding author.

## Abbreviations

CAMSA: Cancer Analytics and Maps for Small Areas
